# Supplementary figures and images for: Env from EIAV vaccine delicately regulates NLRP3 activation via attenuating NLRP3-NEK7 interaction
Source: PLoS Pathog. 2025 Jun 16;21(6):e1012772. doi: 10.1371/journal.ppat.1012772 (PMC12187018; doi:10.1371/journal.ppat.1012772)

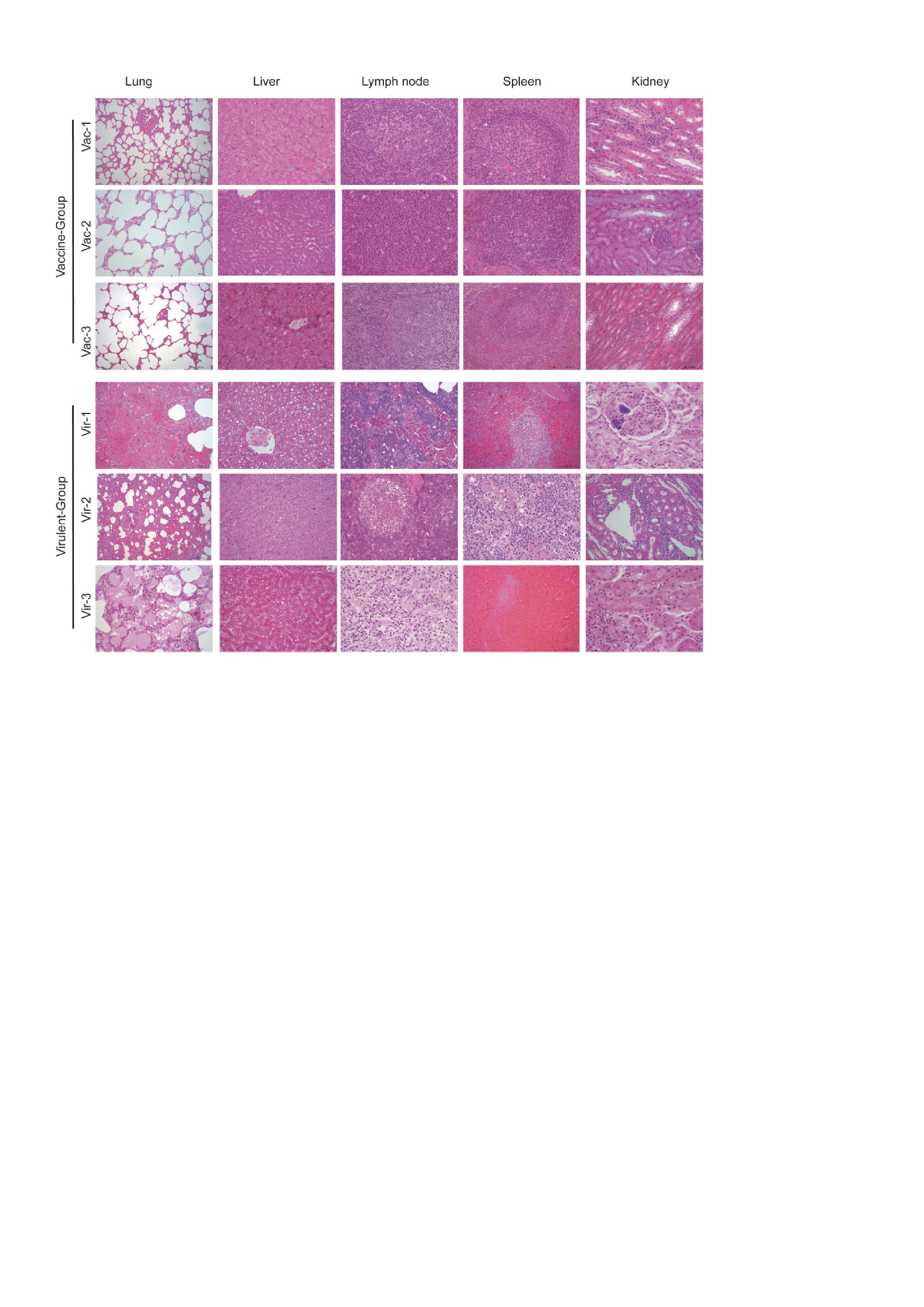

Supplement: S1 Fig — Typical inflammatory pathological changes observed in lung, kidney, liver, spleen and lymph gland on infection with EIAV vaccine or virulent strains are presented separately (haematoxilin and eosin 4 m paraffin sections, original magnification 10x). Severity of pathological lesions and scoring are given in Table 1. (TIF) [file ppat.1012772.s001.tif]

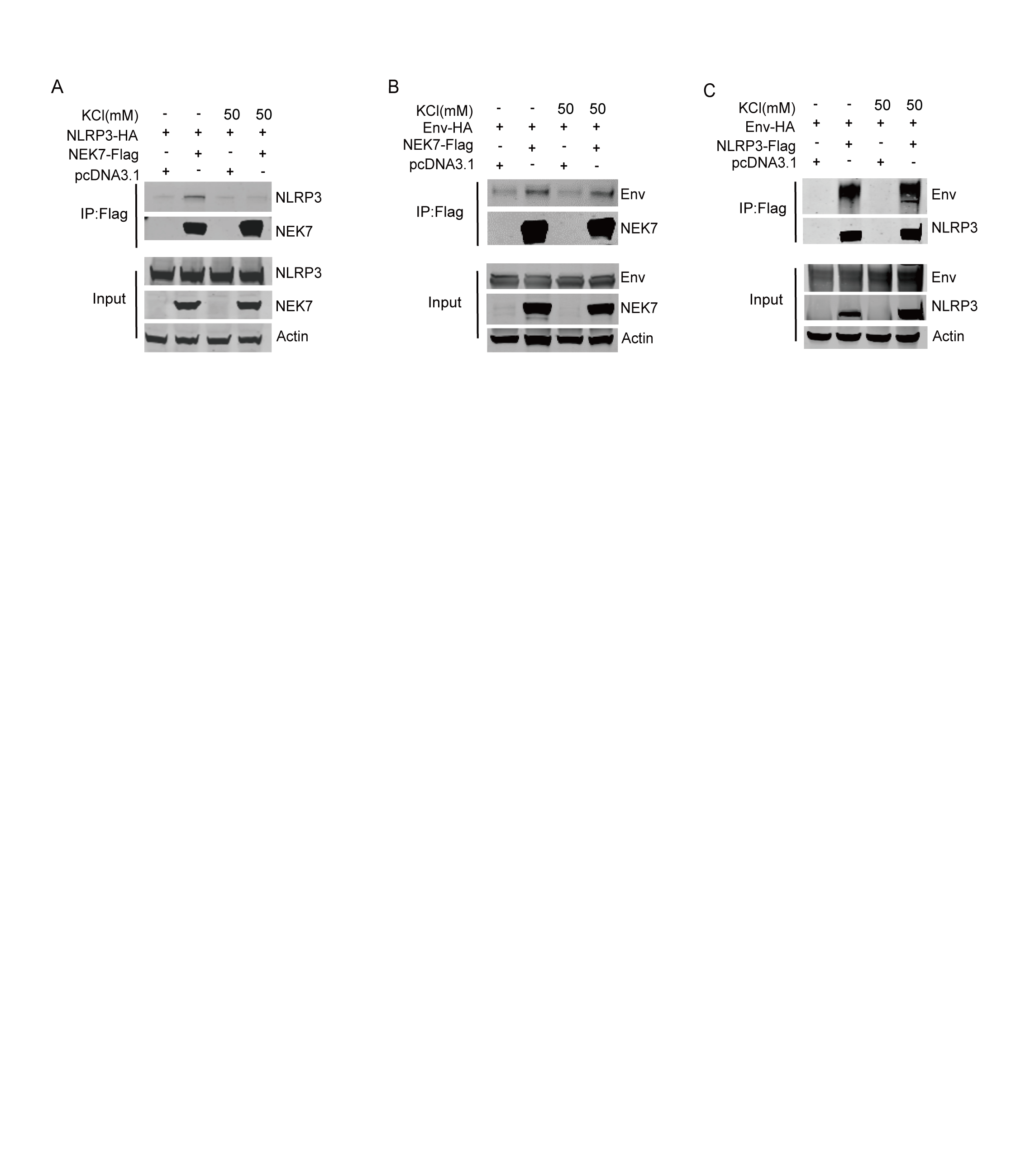

Supplement: S2 Fig — (A) 293T cells were co-transfected with NEK7-Flag and NLRP3-HA in the presence of 50 μM concentrations of KCl (increasing intracellular K+ concentration). Immunoprecipitation and analyses were as described in Fig 3D. (B-C) Procedure was as in (A) but instead of transfection with NEK7 and NLRP3, cells were co-transfected with env-HA and NLRP3-Flag (B) or co-transfected with env-HA and NEK7-Flag (C) at high concentrations of KCl (50 μM). An immunoprecipitation assay was then performed using the indicated antibodies. (TIF) [file ppat.1012772.s002.tif]

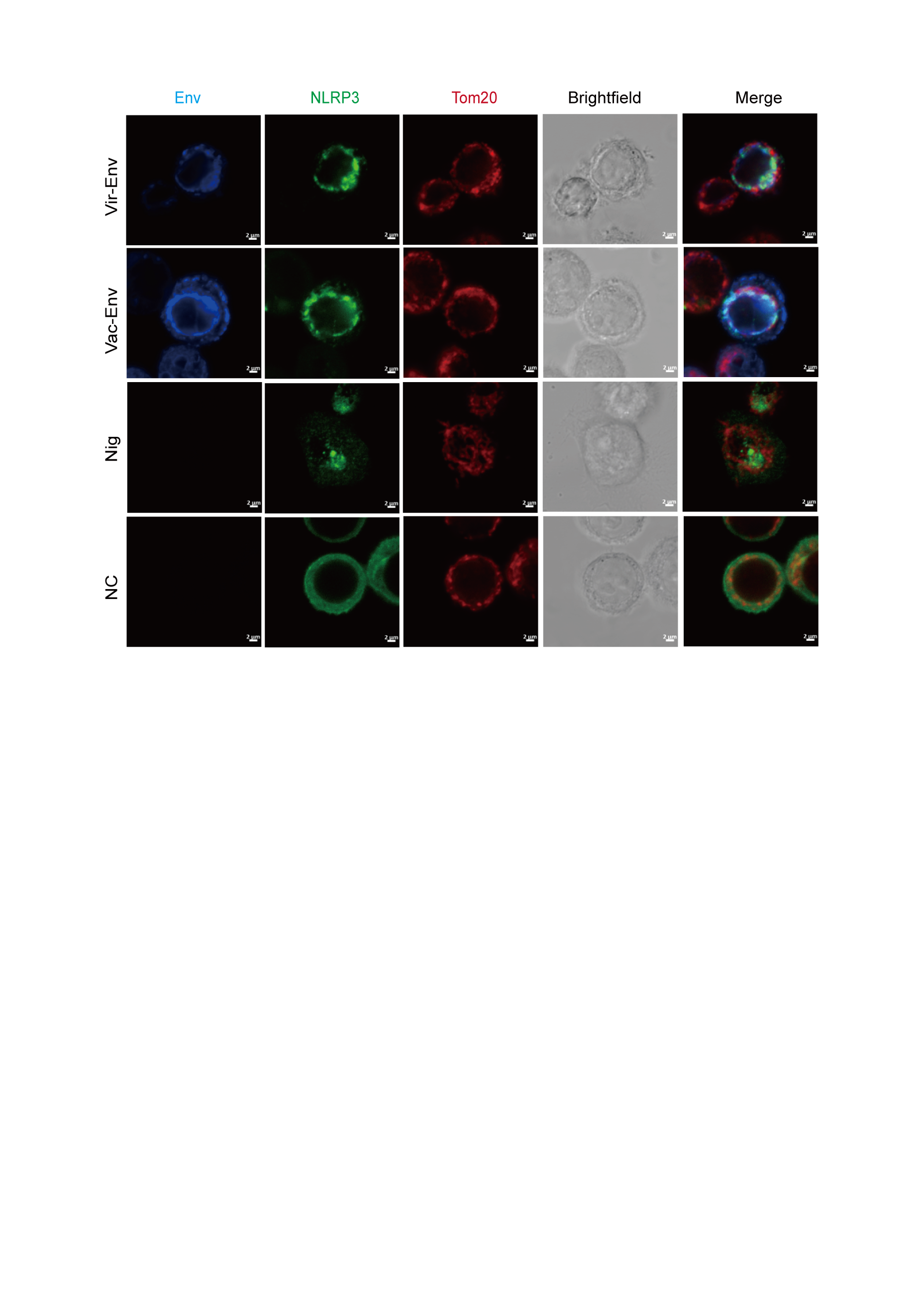

Supplement: S3 Fig — Confocal micrograph of assembly of GFP-NLRP3 and HA-NEK7 not Tom20-labeled mitochondria in 293T cells co-transfected with virulent-env or vaccine-env. (TIF) [file ppat.1012772.s003.tif]

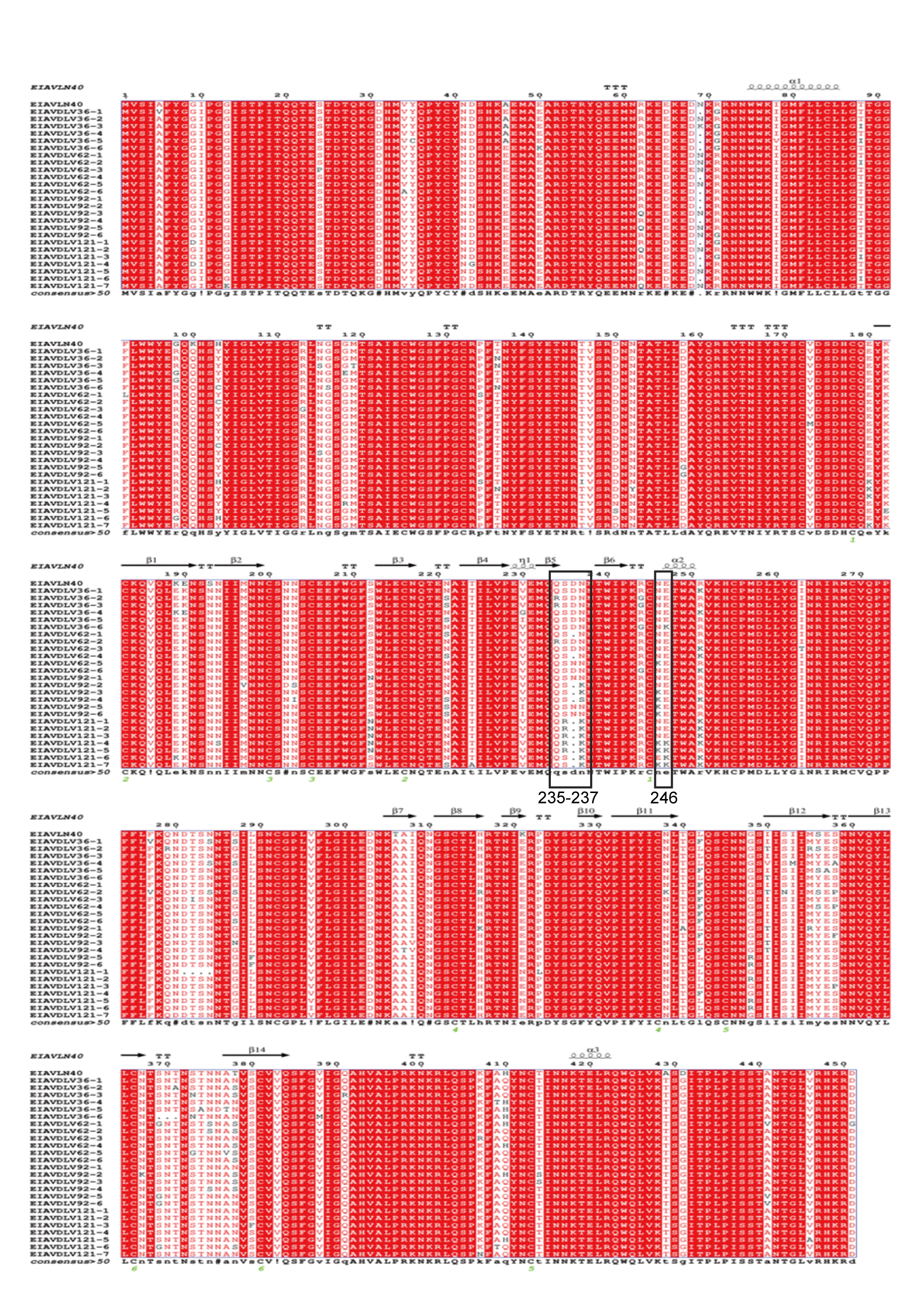

Supplement: S4 Fig — Their conserved mutation sites between the virulent and attenuated vaccine strains were highlighted. (TIF) [file ppat.1012772.s004.tif]
