# Supplementary material for: Env from EIAV vaccine delicately regulates NLRP3 activation via attenuating NLRP3-NEK7 interaction
Source: PLoS Pathog. 2025 Jun 16;21(6):e1012772. doi: 10.1371/journal.ppat.1012772 (PMC12187018; doi:10.1371/journal.ppat.1012772)
Supplement: Attachment — Submitted filename: review response.docx [file ppat.1012772.s007.docx]

**Dear Editor:**

On behalf of all the authors, I would like to express my sincere gratitude to you and the reviewers for providing valuable comments and suggestions on our manuscript. The critiques and suggestions from the reviewers were extremely helpful in improving the quality of this article. We have carefully responded to all the comments point-by-point. The changes in the revised manuscript have been highlighted. We hope these changes will help strengthen our manuscript.

**Editor questions:**

1 Figure 1F shows only one EIAV and not both the virulent and attenuated viruses. Please complete this figure.

**Response:** We thank the reviewer for the valuable comment. We supplemented this result and updated the Figure 1F in the revised manuscript.

2 Please explain how and why the 4 amino acids that are different between the virulent and attenuated virus were selected, how they contribute to virulence, and if additional differences exist.

**Response:** We sincerely apologize for not having elucidated this issue clearly.

In our preliminary research, we assessed the variability of the EIAV *env* during the attenuation process using Bulk PCR and Single Genome Amplification (SGA) methodologies. We found that both the virulent and vaccine strains of EIAV comprise a diverse array of variants, displaying quasispecies characteristics. Notably, multiple mutation sites were identified during the attenuation of the virulent EIAV strain, with further analysis highlighting a consistent pattern of mutation characteristics in these four amino acids. Firstly, we found that mainly these four sites exhibited conserved mutations in the Env protein during the attenuation of the virulent strain, with the mutation frequency showing a gradual and successful accumulation as the passage number increased (S5 Figure). Moreover, the proportion of these combined mutations at sites 235-236, 235-236-237, and 235-236-237-246 gradually increased as the virulence of the strains decreased. Secondly, we observed that the deletion of amino acid 237D led to the loss of an N-glycosylation site (237NNTW240), and that the N237K/N246K mutations were also implicated in the formation of N-glycosylation sites (Liu C, *et al*. Viruses. 2019;11(4). <https://doi.org/10.3390/v11040380>). Changes in glycosylation sites can significantly impact viral virulence. Given the strong correlation between these four sites and viral virulence, this study primarily focuses on these sites. We have already incorporated this content into the revised manuscript (line 277-line 281).

3.Figure 8 in your submission file inventory does not have an in-text citation. If the figure is no longer to be included as part of the submission, please remove it from the file inventory.

**Response:** We apologize for the oversight. Figure 8 (updated to Figure 9 in the revised manuscript) has now been included in the revised manuscript.

4.We have noticed that you have uploaded Supporting Information files, but you have not included a complete list of legends. Please add a full list of legends for your Supporting Information files after the references list.

**Response:** We sincerely apologize for the oversight. We supplemented this content in the revised manuscript.

5. Some material included in your submission may be copyrighted. According to PLOSu2019s copyright policy, authors who use figures or other material (e.g., graphics, clipart, maps) from another author or copyright holder must demonstrate or obtain permission to publish this material under the Creative Commons Attribution 4.0 International (CC BY 4.0) License used by PLOS journals. Please closely review the details of PLOSu2019s copyright requirements here: PLOS Licenses and Copyright. If you need to request permissions from a copyright holder, you may use PLOS's Copyright Content Permission form.

**Response:** Thank you very much for your reminder. We confirm that all materials in the manuscript comply with the copyright policy, and no figures or other materials from other authors have been used.

6. Figure 3. Please confirm whether you drew the images / clip-art within the figure panels by hand. If you did not draw the images, please provide (a) a link to the source of the images or icons and their license / terms of use; or (b) written permission from the copyright holder to publish the images or icons under our CC BY 4.0 license. Alternatively, you may replace the images with opensource alternatives. See these opensource resources you may use to replace images / clip-art:
- https://commons.wikimedia.org
- <https://openclipart.org/>.

**Response:** Thank you very much for your reminder. We confirm that Figure 3A was created by us using Biorender software.

7.Please amend your detailed Financial Disclosure statement. This is published with the article. It must therefore be completed in full sentences and contain the exact wording you wish to be published. State what role the funders took in the study. If the funders had no role in your study, please state: "The funders had no role in study design, data collection and analysis, decision to publish, or preparation of the manuscript.".
If you did not receive any funding for this study, please simply state: u201cThe authors received no specific funding for this work. u201d.

**Response:** Thank you very much for your reminder. The funders had no role in our study. We have amended our detailed Financial Disclosure statement in accordance with your journal's requirements.

**Major Issues**

**Review #1:**

1 The authors state in line 103-104 that the experiment in Fig. 1F confirms that both virulent and attenuated EIAV activate the NLRP3-IL1beta axis, but the figure shows only one EIAV and it is not clear which one is shown. Please provide the additional data.

**Response:** We thank the reviewer for the valuable comment. We have supplemented this result and updated the Figure 1F in the revised manuscript.

2 It is not at all clear to me how the flow cytometry data in Figure 5E can demonstrate the complex formation in contrast to cells just being (more or less) positive for one or both fluorescent labels. Please provide additional data supporting this claim, or provide more quantified data from confocal microscopy, which seems to be the more appropriate method.

**Response:** We sincerely apologize for not employing more rigorous methods to validate this conclusion. We mainly refer to Lee's approach to evaluate whether Env(s) can promote the aggregation of NLRP3 and NEK7 (Autophagy 2022 May;18(5):990-1004. DOI:[10.1080/15548627.2021.1965426](https://www.x-mol.com/paperRedirect/1435095582129319936)). The evaluation of colocalization between fluorescently labeled proteins is primarily determined using the Pearson correlation coefficient, which is computed by analyzing the fluorescence intensities of the two labeled proteins within the gated cell population. We have annotated the original images and calculated the corresponding ratios (updated to S4 Figure). To further validate these findings, we utilized the Bimolecular Fluorescence Complementation (BiFC) assay. This method allows for the reconstitution of a fluorescent protein upon interaction between the two target proteins, enabling the detection of their binding affinity by measuring the mean fluorescence intensity using flow cytometry. Our results support the original conclusions. These data have been incorporated into Figure 5E and 5F in the revised manuscript.

**Review #2:**

1.authors try to display the differences of inflammatory response and host responses between the attenuated vaccine and its virulent mother strain. However, the results seem to show that the virulence play important role in activate the NLRP3-IL-1β. The EIAV-Env of the attenuated vaccine or its virulent mother strain looks like to play the similar role in this study.

**Response:** We sincerely apologize for not having addressed this issue clearly in our previous communication. Our research has demonstrated that the Env(s) of both the virulent and vaccine strains of EIAV modulate NLRP3 inflammasome activation by interacting with P2X7 (R), NLRP3, and NEK7. Importantly, the binding affinity of Env(s) from the virulent and attenuated strains to these proteins did not show significant differences. Mechanistically, the observed variation in NLRP3 activation between the virulent and attenuated EIAV strains is primarily due to the differential ability of their respective Env to facilitate the binding between NLRP3 and NEK7. Through Co-IP assay, we further identified four key conserved mutations in the vaccine-Env as the main contributors to the distinct NLRP3-NEK7 interactions observed between the virulent and vaccine strains. Structural prediction analysis reveal that four key mutations cause the vaccine-Env to form a salt bridge, altering the surface charge distribution. These changes lead to conformational shifts in the loop region of the corresponding domain in the virulent-Env (These data are presented in Figure 8A and 8B). It is speculated that, compared to the virulent-Env, the vaccine-Env exhibits a reduced ability to bind with other proteins. Based on these findings, our study reveals that these four conserved virulence-associated mutations play a critical role in modulating the activation level of the NLRP3 inflammasome through their impact on the binding affinity between NLRP3 and NEK7. We have supplied this result in the revised manuscript (line 296-line 308).

2.Env was the main factor affecting the effects of the NLRP3-IL-1β-axis activation induced by EIAV infection. Interestingly, no disparity between the abilities of Env to bind these proteins was observed between the virulent and vaccine EIAV strains. Notably, no differences were observed in the Env-mediated K^+^ efflux between the two EIAV strains. At the end, authors conclude that four amino acid residues in Env contribute to the differential regulation of the NLRP3-IL-1β-axis. Authors did not discuss whether these amino acid residues influence the virulence or not.

**Response:** We thank the reviewer for the valuable comment. As the construction of an infectious clone incorporating these four critical amino acid mutations within the virulent strain backbone has not yet been achieved, it is currently not feasible to directly assess the specific effects of these sites on viral virulence in equine models. However, based on our preliminary studies, we speculated that these four critical amino acids are closely associated with the virulence of EIAV: 1) During the attenuation process from the virulent strain to the vaccine strain, despite the presence of random mutations, mainly these four sites exhibit stable mutational characteristics. Statistical analysis indicates a significant correlation between the mutation frequency at these sites and changes in viral virulence (Liu C, *et al*. Viruses. 2019;11(4)); 2) The mutations at these four sites directly result in the loss of two glycosylation sites on the Env protein of the vaccine strain, thereby influencing viral pathogenicity. Furthermore, our findings demonstrate that these four mutations are the primary cause of the differential regulation of NLRP3 inflammasome activity between the virulent and attenuated EIAV strains, further supporting the conclusion that these amino acid residues are closely associated with EIAV virulence. We have systematically elaborated on this in the discussion section (line 277-line 281, line 363-line 368).

3 Authors should display whether four amino acid residues in Env contribute to the virulence.

**Response:** We sincerely appreciate the reviewer’s valuable comment and apologize for the insufficient detail provided in our initial submission. Our preliminary studies have demonstrated that the attenuation of EIAV involves multiple site-specific mutations. Through comprehensive sequence alignment analysis, we identified that mutations at these four specific sites exhibit a significant correlation with alterations in viral virulence (Liu C, *et al*. Viruses. 2019;11(4)). Notably, only the deletion at position D236 serves as a molecular marker distinguishing virulent from attenuated EIAV strains (Liu C, *et al*. Viruses. 2019;11(4); Han X, *et al*. Viral Immunol. 2010;23(5)). Furthermore, we observed that the deletion of amino acid 237D results in the loss of a critical N-glycosylation motif (237NNTW240). Additionally, the N237K and N246K mutations were found to be associated with the formation of novel N-glycosylation sites, which may play a role in modulating viral pathogenicity. These findings strongly suggest that four amino acid residues are closely linked to the virulence of EIAV. We have included additional details regarding these observations in the revised manuscript (S5 and line 275 -line 279).

**Minor Issues:**

**Reviewer #1:**

1.The figures show a lot of bar charts and it is not clear how many data points were collected, and in how many independent experiments. Please provide this information in all figure legends.

**Response:** We appreciate the reviewer’s comment and have provided the relevant information in the revised manuscript.

2. line 122: please add this statistical difference to the figure.

**Response:** We thank the reviewer for the valuable comment. We have added this information and updated the Figure 2F.

3. line 154: please avoid the abbreviation "Gly", you only use it two times in the text anyway and it makes it harder to understand for the reader

**Response:** We appreciate the reviewer’s comment. We have replaced 'Gly' with 'Glybenclamide' in the revised manuscript.

4.line 193: the authors mention "four key amino acids", they should elaborate whether these are the only different amino acids in the two EIAV Env sequences, or whether they picked them because of some special characteristics? in line 263, the authors mention a high diversity in Env of the EIAV vaccine strain, which implies that more than these 4 amino acids are different. Show in an alignment of the whole Env (Gp90) sequences might be helpful. Furthermore, it would help the reader if the authors could provide insight about the location of these four amino acids in the 3D structure of the Env protein, ideally in a figure/schematic.

**Response:** We extend our gratitude to the reviewer for their insightful comment. As you correctly surmised, the attenuation process of the virulent EIAV strain involves mutations at multiple sites. We focused on these four specific sites for the following reasons: 1) Mainly these four sites exhibited conserved mutations in the Env during the attenuation of the virulent strain, with the mutation frequency demonstrating a gradual and consistent accumulation as the passage number increased. 2) Mutations at these four sites resulted in the loss of N-glycosylation sites. These findings strongly suggest that these sites are closely associated with the virulence of EIAV. We have supplemented the sequence alignment data for the Env (Gp90) (S5 Figure), which enhances the clarity and depth of our analysis.

According to the reviewer’s recommendation, we further employed AlphaFold 2 to predict the three-dimensional structures of the Env (s) for both virulent and attenuated strains. The analysis not only underscores the critical functional significance of these four amino acids but also demonstrates that mutations at these sites induce a shift in the surface charge distribution of the virulent strain from predominantly negative to positive. Notably, the R235 mutation forms a salt bridge with E246, located in a proximal region, resulting in substantial conformational rearrangements within the groove (Figure 8A and 8B). This structural perturbation is likely to impair the binding affinity of the vaccine strain’s Env to interacting partners, providing mechanistic insights into our experimental observations. These findings have been comprehensively integrated into the revised manuscript to enhance the clarity and depth of our conclusions (line 275-line 279 and line 296 -line 308).

5. line 254-256: "N246 mutation in wt" -> please rephrase so that it is easier to understand which sequence carries which amino acid.

**Response:** We apologize for this confusing. We have delated this sentence in the revised manuscript.

6. line 271-273: the authors need to provide more context for the statement that EIAV "evolves flexibly rather than lethally", it is not clear what they mean.

**Response:** We apologize for this confusing. We have already updated this part in the discussion (line 385-line 388).

7. To make the fluorescene images easier to interpret, please add brightfield images.

**Response:** We appreciate the reviewer’s comment. We have updated the Figure 1E in the revised manuscript.

8. Please avoid the speculation that a similar approach would lead to an HIV vaccine, this is a very big stretch.

**Response:** We would like to express our gratitude to the reviewer for pointing out this issue. The mentioned similar sentences have been eliminated in the revised version of our manuscript.

9. Methods section: please make sure that enough details are provided so that results can be replicated, e.g. how much plasmid DNA was used for transfections, how many cells were infected with 2x10^5 TCID50 (->MOI)?
lines 464-466: the authors only mention labeled secondary antibodies, what primary antibodies were used?
**Response:** We sincerely apologize for omitting these details in the initial draft. We have now supplemented them based on the reviewer's suggestions.

10. line 472: the authors used a Student's t test for the statistical analysis, but this is only appropriate for the comparison of two groups. If comparing more groups, use either an ANOVA or perform an adjustment for multiple comparisons.
**Response:** We sincerely apologize for the inadvertent oversight in our initial selection of statistical methods for data analysis. Upon careful re-evaluation using the appropriate statistical approach, we have confirmed that the results remain consistent with our original findings. The manuscript has been revised accordingly to reflect this updated analysis, ensuring the accuracy and reliability of our conclusions.

11. line 473: the authors state that the error bars in the figures show the "SD (standard error of the mean)". SD = standard deviation and SEM = standard error of the mean are not the same and not interchangeable, they should use the standard deviation and modify the figures accordingly.

**Response:** We sincerely apologize for the previous error and have made the necessary corrections in the revised manuscript. All original data included were generated using standard deviation (SD) for calculation (line 629-line 631).
