# Supplementary material for: Env from EIAV vaccine delicately regulates NLRP3 activation via attenuating NLRP3-NEK7 interaction
Source: PLoS Pathog. 2025 Jun 16;21(6):e1012772. doi: 10.1371/journal.ppat.1012772 (PMC12187018; doi:10.1371/journal.ppat.1012772)
Supplement: Attachment — Submitted filename: response to reviews.docx [file ppat.1012772.s008.docx]

**Review #1:**

1 The authors have performed a convincing new experiment using bimolecular fluorescence complementation to prove the colocalization of NEK7 and NLRP3. The previous experiment where they tried to prove the colocalization by flow cytometry should be removed altogether from text and supplement, as it is technically not valid. The cited literature shows a similar dot plot, but l believe they are showing different data in these plots (confocal microscopy data). it is not technically plausible to use flow cytometry- unless itis imaging flow cytometry - to study colocalization. Again, l highly appreciated the bimolecular fluorescence complementation study, which is convincing and should be the only result shown in the revised manuscript

**Response:** We thank the reviewer for the valuable comment. We have deleted this result in the revised manuscript.

2. The authors have added information about the number of experiments to this revised manuscript but there is still no information about the number of data points that are shown in the bar graphs.

**Response:** We sincerely thank the reviewer for their valuable comment. As suggested, we have supplemented the necessary information in the revised manuscript to address this point.

3. Please add brightfield images to all figures showing flourescence microscopy data, at the moment they have only been added to Figure 1E and Figure 5E.

**Response:** We thank the reviewer for the valuable comment. We have supplemented these results and updated the Figure 1F, Figure 2D, Figure3 G-I, Figure 6 and Figure S3 in the revised manuscript.

4. In the Methods section "Infection", there is still no information about the number of cells that were infected with 2x10^5TCID50.

**Response:** We sincerely apologize for the insufficient detail provided in our initial submission. We have supplemented this information in the revised manuscript (line 464).
